# 5-(2-Bromo­phen­yl)-1,3,4-thia­diazol-2-amine

**DOI:** 10.1107/S1600536808027827

**Published:** 2008-09-06

**Authors:** Li-He Yin, Rong Wan, Yao Wang, Feng Han, Jin-Tang Wang

**Affiliations:** a Department of Applied Chemistry, College of Science, Nanjing University of Technology, No.5 Xinmofan Road, Nanjing 210009, People’s Republic of China

## Abstract

In the title compound, C_8_H_6_BrN_3_S, the thia­diazole ring is oriented at a dihedral angle of 48.35 (3)° with respect to the bromo­phenyl ring. In the crystal structure, inter­molecular N—H⋯N hydrogen bonds link the mol­ecules.

## Related literature

For related literature, see: Nakagawa *et al.* (1996 ▶); Omar *et al.* (1986 ▶); Wang *et al.* (1999 ▶). For bond-length data, see: Allen *et al.* (1987 ▶).
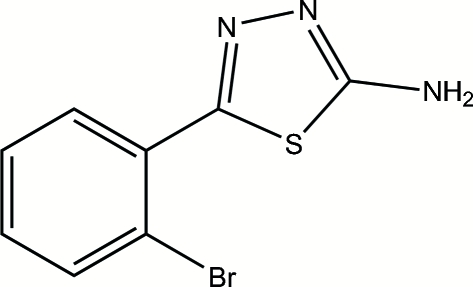


## Experimental

### 

#### Crystal data

C_8_H_6_BrN_3_S
*M* *_r_* = 256.13
Monoclinic, 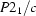

*a* = 14.869 (3) Å
*b* = 8.0250 (16) Å
*c* = 7.9480 (16) Å
β = 97.43 (3)°
*V* = 940.4 (3) Å^3^
*Z* = 4
Mo *K*α radiation
μ = 4.54 mm^−1^
*T* = 298 (2) K
0.30 × 0.10 × 0.10 mm

#### Data collection

Enraf–Nonius CAD-4 diffractometer
Absorption correction: ψ scan (North *et al.*, 1968 ▶) *T*_min_ = 0.343, *T*_max_ = 0.659
1832 measured reflections
1694 independent reflections
972 reflections with *I* > 2σ(*I*)
*R*_int_ = 0.034
3 standard reflections frequency: 120 min intensity decay: none

#### Refinement

*R*[*F* ^2^ > 2σ(*F* ^2^)] = 0.060
*wR*(*F* ^2^) = 0.153
*S* = 0.97
1694 reflections
118 parameters
H-atom parameters constrained
Δρ_max_ = 0.41 e Å^−3^
Δρ_min_ = −0.57 e Å^−3^

### 

Data collection: *CAD-4 Software* (Enraf–Nonius, 1989 ▶); cell refinement: *CAD-4 Software*; data reduction: *XCAD4* (Harms & Wocadlo, 1995 ▶); program(s) used to solve structure: *SHELXS97* (Sheldrick, 2008 ▶); program(s) used to refine structure: *SHELXL97* (Sheldrick, 2008 ▶); molecular graphics: *SHELXTL* (Sheldrick, 2008 ▶); software used to prepare material for publication: *SHELXTL*.

## Supplementary Material

Crystal structure: contains datablocks global, I. DOI: 10.1107/S1600536808027827/hk2522sup1.cif

Structure factors: contains datablocks I. DOI: 10.1107/S1600536808027827/hk2522Isup2.hkl

Additional supplementary materials:  crystallographic information; 3D view; checkCIF report

## Figures and Tables

**Table 1:** Hydrogen-bond geometry (Å, °)

*D*—H⋯*A*	*D*—H	H⋯*A*	*D*⋯*A*	*D*—H⋯*A*
N3—H3*A*⋯N1^i^	0.86	2.27	3.092 (7)	160
N3—H3*A*⋯N2^i^	0.86	2.61	3.221 (7)	129
N3—H3*B*⋯N2^ii^	0.86	2.06	2.896 (7)	163

Symmetry codes: (i) 
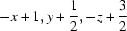
; (ii) 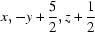.

**Fig. 1.**
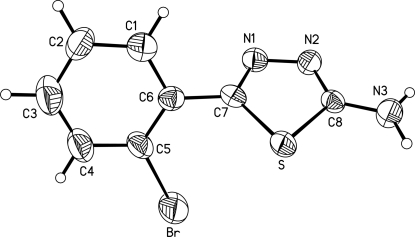
The molecular structure of the title molecule, with the atom-numbering scheme. Displacement ellipsoids are drawn at the 50% probability level.

**Fig. 2.**
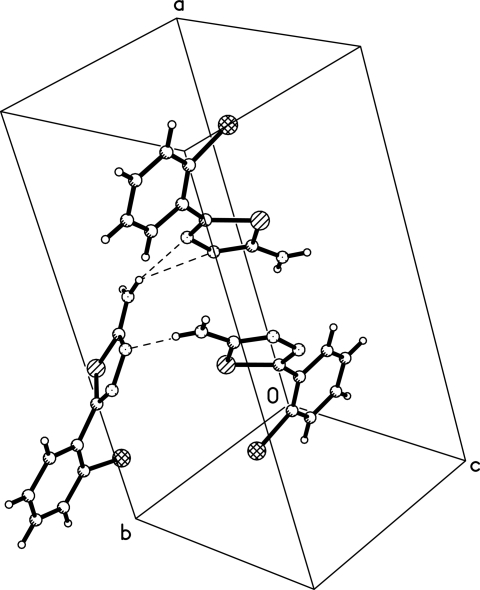
A partial packing diagram of the title compound. Hydrogen bonds are shown as dashed lines.

## supplementary crystallographic information

### Comment

1,3,4-Thiadiazole derivatives represent an interesting class of compounds possessing broad spectrum biological activities (Nakagawa *et al.*, 1996). These compounds are known to exhibit diverse biological effects, such as insecticidal and fungicidal activities (Wang *et al.*,  1999). It can also be widely used in the field of medicine, such as anti-cancer drugs (Omar *et al.*, 1986).

In the molecule of the title compound, (Fig. 1), the bond lengths (Allen *et al.*, 1987) and angles are generally within normal ranges. Rings A (C1–C6) and B (S/N1/N2/C7/C8) are, of course, planar, and they are oriented at a dihedral angle of 48.35 (3)°.

In the crystal structure, intermolecular N—H···N hydrogen bonds (Table 1) link the molecules (Fig. 2), in which they may be effective in the stabilization of the structure.

### Experimental

For the preparation of the title compound, 2-bromobenzoic acid (5 mmol) and thiosemicarbazide (5 mmol) were added in toluene (50 ml), which is heated under reflux for 4 h. The reaction mixture was left to cool to room temperature, poured into ice water, filtered, and the filter cake was crystallized from acetone to give title compound (m.p. 486–487 K). Crystals suitable for X-ray analysis were obtained by slow evaporation of an acetone solution.

### Refinement

H atoms were positioned geometrically, with N—H = 0.86 Å (for NH_2_) and C—H = 0.93 Å for aromatic H, respectively, and constrained to ride on their parent atoms with U_iso_(H) = 1.2U_eq_(C,N).

### Crystal data

**Table:**

C_8_H_6_BrN_3_S	*F*(000) = 504
*M**_r_* = 256.13	*D*_x_ = 1.809 Mg m^−^^3^
Monoclinic, *P*2_1_/*c*	Melting point = 486–487 K
Hall symbol: -P 2ybc	Mo *K*α radiation, λ = 0.71073 Å
*a* = 14.869 (3) Å	Cell parameters from 25 reflections
*b* = 8.0250 (16) Å	θ = 10–14°
*c* = 7.9480 (16) Å	µ = 4.55 mm^−^^1^
β = 97.43 (3)°	*T* = 298 K
*V* = 940.4 (3) Å^3^	Block, colorless
*Z* = 4	0.30 × 0.10 × 0.10 mm

### Data collection

**Table:**

Enraf–Nonius CAD-4 diffractometer	972 reflections with *I* > 2σ(*I*)
Radiation source: fine-focus sealed tube	*R*_int_ = 0.034
graphite	θ_max_ = 25.2°, θ_min_ = 1.4°
ω/2θ scans	*h* = −17→17
Absorption correction: ψ scan (North *et al.*, 1968)	*k* = 0→9
*T*_min_ = 0.343, *T*_max_ = 0.659	*l* = 0→9
1832 measured reflections	3 standard reflections every 120 min
1694 independent reflections	intensity decay: none

### Refinement

**Table:**

Refinement on *F*^2^	Primary atom site location: structure-invariant direct methods
Least-squares matrix: full	Secondary atom site location: difference Fourier map
*R*[*F*^2^ > 2σ(*F*^2^)] = 0.060	Hydrogen site location: inferred from neighbouring sites
*wR*(*F*^2^) = 0.153	H-atom parameters constrained
*S* = 0.97	*w* = 1/[σ^2^(*F*_o_^2^) + (0.0838*P*)^2^] where *P* = (*F*_o_^2^ + 2*F*_c_^2^)/3
1694 reflections	(Δ/σ)_max_ < 0.001
118 parameters	Δρ_max_ = 0.41 e Å^−^^3^
0 restraints	Δρ_min_ = −0.57 e Å^−^^3^

### Special details

**Table:**

Geometry. All e.s.d.'s (except the e.s.d. in the dihedral angle between two l.s. planes) are estimated using the full covariance matrix. The cell e.s.d.'s are taken into account individually in the estimation of e.s.d.'s in distances, angles and torsion angles; correlations between e.s.d.'s in cell parameters are only used when they are defined by crystal symmetry. An approximate (isotropic) treatment of cell e.s.d.'s is used for estimating e.s.d.'s involving l.s. planes.
Refinement. Refinement of *F*^2^ against ALL reflections. The weighted *R*-factor *wR* and goodness of fit *S* are based on *F*^2^, conventional *R*-factors *R* are based on *F*, with *F* set to zero for negative *F*^2^. The threshold expression of *F*^2^ > σ(*F*^2^) is used only for calculating *R*-factors(gt) *etc*. and is not relevant to the choice of reflections for refinement. *R*-factors based on *F*^2^ are statistically about twice as large as those based on *F*, and *R*- factors based on ALL data will be even larger.

### Fractional atomic coordinates and isotropic or equivalent isotropic displacement parameters (Å^2^)

**Table:**

	*x*	*y*	*z*	*U*_iso_*/*U*_eq_	
Br	0.88329 (5)	1.09232 (10)	1.09121 (14)	0.0845 (5)	
S	0.65602 (11)	1.1146 (2)	1.0932 (2)	0.0458 (5)	
N1	0.6259 (3)	0.9573 (7)	0.8134 (6)	0.0424 (13)	
N2	0.5587 (3)	1.0773 (6)	0.8048 (6)	0.0430 (13)	
N3	0.5081 (3)	1.2909 (6)	0.9666 (6)	0.0480 (14)	
H3A	0.4635	1.3143	0.8901	0.058*	
H3B	0.5158	1.3467	1.0597	0.058*	
C1	0.7308 (5)	0.6693 (8)	0.9656 (9)	0.0512 (18)	
H1	0.6722	0.6431	0.9176	0.061*	
C2	0.7921 (5)	0.5423 (8)	1.0064 (10)	0.0591 (19)	
H2	0.7750	0.4319	0.9866	0.071*	
C3	0.8787 (5)	0.5816 (10)	1.0769 (10)	0.068 (2)	
H3	0.9204	0.4968	1.1067	0.081*	
C4	0.9041 (4)	0.7438 (10)	1.1036 (9)	0.059 (2)	
H4	0.9630	0.7698	1.1506	0.071*	
C5	0.8416 (4)	0.8692 (8)	1.0602 (9)	0.0493 (17)	
C6	0.7542 (4)	0.8348 (7)	0.9941 (8)	0.0378 (14)	
C7	0.6811 (4)	0.9618 (8)	0.9507 (7)	0.0363 (14)	
C8	0.5647 (4)	1.1688 (7)	0.9412 (7)	0.0332 (14)	

### Atomic displacement parameters (Å^2^)

**Table:**

	*U*^11^	*U*^22^	*U*^33^	*U*^12^	*U*^13^	*U*^23^
Br	0.0417 (5)	0.0576 (5)	0.1483 (10)	−0.0105 (4)	−0.0096 (5)	−0.0066 (6)
S	0.0396 (9)	0.0538 (10)	0.0394 (9)	0.0102 (8)	−0.0131 (7)	−0.0097 (8)
N1	0.042 (3)	0.046 (3)	0.037 (3)	0.000 (3)	−0.001 (3)	−0.003 (3)
N2	0.045 (3)	0.043 (3)	0.037 (3)	0.008 (3)	−0.008 (2)	−0.005 (3)
N3	0.049 (3)	0.056 (4)	0.036 (3)	0.015 (3)	−0.008 (2)	−0.007 (3)
C1	0.046 (4)	0.049 (4)	0.057 (5)	−0.003 (3)	0.005 (3)	0.001 (4)
C2	0.057 (4)	0.035 (4)	0.086 (5)	0.004 (3)	0.013 (4)	0.004 (4)
C3	0.050 (4)	0.068 (6)	0.083 (6)	0.021 (4)	0.005 (4)	0.012 (5)
C4	0.037 (4)	0.064 (5)	0.073 (5)	0.009 (4)	−0.003 (4)	0.009 (4)
C5	0.031 (3)	0.049 (4)	0.065 (4)	−0.004 (3)	−0.004 (3)	0.006 (3)
C6	0.038 (3)	0.040 (3)	0.036 (3)	−0.002 (3)	0.006 (3)	−0.004 (3)
C7	0.029 (3)	0.042 (3)	0.037 (3)	−0.006 (3)	0.001 (3)	0.001 (3)
C8	0.029 (3)	0.035 (3)	0.034 (4)	0.000 (3)	−0.003 (3)	0.004 (3)

### Geometric parameters (Å, °)

**Table:**

Br—C5	1.901 (7)	C3—H3	0.9300
N1—N2	1.383 (7)	C4—C5	1.383 (9)
N3—H3A	0.8600	C4—H4	0.9300
N3—H3B	0.8600	C5—C6	1.365 (8)
C1—C2	1.377 (9)	C6—C7	1.498 (8)
C1—C6	1.384 (8)	C7—N1	1.278 (7)
C1—H1	0.9300	C7—S	1.742 (6)
C2—C3	1.373 (10)	C8—N2	1.302 (7)
C2—H2	0.9300	C8—N3	1.325 (7)
C3—C4	1.365 (11)	C8—S	1.752 (6)
			
C2—C1—C6	121.8 (7)	C5—C6—C1	117.6 (6)
C2—C1—H1	119.1	C5—C6—C7	125.3 (6)
C6—C1—H1	119.1	C1—C6—C7	117.1 (6)
C3—C2—C1	118.9 (7)	N1—C7—C6	123.0 (6)
C3—C2—H2	120.5	N1—C7—S	114.0 (5)
C1—C2—H2	120.5	C6—C7—S	122.6 (4)
C4—C3—C2	120.6 (7)	N2—C8—N3	124.6 (5)
C4—C3—H3	119.7	N2—C8—S	113.4 (4)
C2—C3—H3	119.7	N3—C8—S	122.0 (5)
C3—C4—C5	119.4 (6)	C7—N1—N2	113.7 (5)
C3—C4—H4	120.3	C8—N2—N1	112.4 (5)
C5—C4—H4	120.3	C8—N3—H3A	120.0
C6—C5—C4	121.6 (6)	C8—N3—H3B	120.0
C6—C5—Br	121.2 (5)	H3A—N3—H3B	120.0
C4—C5—Br	117.1 (5)	C7—S—C8	86.4 (3)
			
C6—C1—C2—C3	−0.2 (11)	C1—C6—C7—N1	−44.3 (9)
C1—C2—C3—C4	−1.0 (12)	C5—C6—C7—S	−51.2 (8)
C2—C3—C4—C5	0.5 (12)	C1—C6—C7—S	128.4 (6)
C3—C4—C5—C6	1.3 (11)	C6—C7—N1—N2	174.6 (5)
C3—C4—C5—Br	−177.2 (6)	S—C7—N1—N2	1.3 (7)
C4—C5—C6—C1	−2.5 (11)	N3—C8—N2—N1	−179.0 (6)
Br—C5—C6—C1	176.0 (5)	S—C8—N2—N1	0.6 (6)
C4—C5—C6—C7	177.2 (6)	C7—N1—N2—C8	−1.2 (7)
Br—C5—C6—C7	−4.4 (9)	N1—C7—S—C8	−0.8 (5)
C2—C1—C6—C5	1.9 (11)	C6—C7—S—C8	−174.1 (5)
C2—C1—C6—C7	−177.8 (6)	N2—C8—S—C7	0.1 (5)
C5—C6—C7—N1	136.1 (7)	N3—C8—S—C7	179.6 (5)

### Hydrogen-bond geometry (Å, °)

**Table:**

*D*—H···*A*	*D*—H	H···*A*	*D*···*A*	*D*—H···*A*
N3—H3A···N1^i^	0.86	2.27	3.092 (7)	160.
N3—H3A···N2^i^	0.86	2.61	3.221 (7)	129.
N3—H3B···N2^ii^	0.86	2.06	2.896 (7)	163.

Symmetry codes: (i) −*x*+1, *y*+1/2, −*z*+3/2; (ii) *x*, −*y*+5/2, *z*+1/2.
